# Immunomodulatory Effects of *Lepidium meyenii* Walp. Polysaccharides on an Immunosuppression Model Induced by Cyclophosphamide

**DOI:** 10.1155/2022/1210890

**Published:** 2022-07-04

**Authors:** Wen-ting Fei, Na Yue, Ai-min Li, Shu-hui Yu, Dan-ping Zhao, Ying-li Zhu, Chun Wang, Jian-jun Zhang, Lin-yuan Wang

**Affiliations:** ^1^School of Chinese Materia Medica, Beijing University of Chinese Medicine, China; ^2^School of Traditional Chinese Medicine, Beijing University of Chinese Medicine, China; ^3^New Era Health Industry (Group) Co., Ltd, China

## Abstract

**Background:**

*Lepidium meyenii* Walp. (Maca) has emerged as a functional plant food and traditional herb owing to its biological activities; Maca polysaccharides as an important active component of Maca have good immunomodulatory effect; however, studies on the immunomodulatory effect of Maca polysaccharides are mainly focused on macrophages; little attention has been devoted to the mechanisms and other immune cells. This study is aimed at investigating the immunomodulatory effects and mechanisms of Maca polysaccharides.

**Methods:**

Sixty mice were divided into five groups, and the mice were injected with cyclophosphamide to establish an immunosuppression model except for those in the common group. The body weights were measured, as well as immune-related indices, such as organ indices, haematological parameters, lymphocyte cycle, and proliferation, cytokine, and protein expression levels.

**Results:**

The weight loss and immune organ index decline caused by cyclophosphamide could be reversed by MP. Furthermore, MP increased WBC and HGB counts and reduced the ratio of G0/G1 phase obviously, increased the proportion of S phase and G2/M phase in peripheral blood lymphocytes, increased the counts of CD4+ T cells and the ratio of CD4+/CD8+, and reduced the inhibition rate of splenic lymphocytes. MP affected the production of cytokines by increasing IFN-*γ*, TNF-*α*, and IL-2 levels and by decreasing IL-4 levels. MP increased the mRNA expression of T-bet and the protein expression of Bcl-2 in the spleen and decreased the protein expression of caspase-3 and Bax.

**Conclusions:**

Maca polysaccharides might be the basic material for Maca's immunomodulatory effect. The mechanism was perhaps related to inhibiting lymphocyte apoptosis and promoting the balance of Th1/Th2 cell subsets.

## 1. Introduction

*Lepidium meyenii* Walp. (Maca) is a plant native to the Andean region of Peru and belongs to the Brassicaceae family [1]. It has been cultivated for at least 2000 years and generally grows in high-altitude areas characterized by extreme weather conditions, such as rocky soil, intense sunlight, and windy atmosphere [2, 3]. The root is the main edible part of Maca, and its bioactive components that benefit the human body have been isolated, such as phytosterols, nonstarch polysaccharides, polyphenols, macamides, and glucosinolates [4]. Scientific evidence has shown that Maca's effects include sexual dysfunction regulation [5], neuroprotection, improvement of memory and learning [6], antidepressant, antioxidant [7], immunomodulatory effect on macrophages [8], anti-inflammatory activities, and skin protection. Maca was introduced into China in 2011 and is cultivated widely in Yunnan and Tibet in China for its beneficial biological activities. Currently, Maca is widely used in the fields of medicine and health to protect our bodies. We checked the website of the State Administration for Market Regulation of China (http://ypzsx.gsxt.gov.cn/specialfood/#/food) and found that there are 56 health care products with Maca as the main raw material. Notably, 40 of the products had Maca mixed with other traditional Chinese medicines, such as American ginseng and epimedium, to relieve physical fatigue and enhance immunity.

As an important component of Maca, Maca polysaccharides (MP) are composed of galactose, rhamnose, arabinose, glucose, xylose, fucose, mannose, and so on [9]. The differences in the compositions and percentages of polysaccharides are attributed to temperature changes, column chromatography, and analytical methods [10, 11]. Many natural polysaccharides are potential candidates for use as immunomodulators with wide applications because of their immunostimulatory activity [12]. Maca polysaccharides are one of the main effective components of Maca and can be explored as sources of bioactive compounds [13]. Studies have showed that Maca polysaccharides have a good effect on antifatigue [14], endogenous antioxidant [7], and energy metabolism [15]. In addition, Maca polysaccharides have good immune-regulatory activity on macrophages by regulating the polarization and increasing the phagocytosis [16]. It is worth noting that studies on the immunomodulatory effect of Maca polysaccharides are mostly about macrophages, but few are about the whole body state.

Cyclophosphamide (CYP) is a commonly used chemotherapeutic agent for treating various cancers and autoimmune disorders [17, 18]. At the same time, it also led to weakened immune function in normal cells while fighting cancer cells. Thus, intraperitoneal cyclophosphamide injection is one of the most appropriate methods to establish an immunosuppression model. The immune system is divided into innate and adaptive immune systems based on their functions. The innate immune systems initiate primary defence reactions and fight infectious agents by inducing an inflammatory response, which involves natural killer cells, mast cells, phagocytes, macrophages, monocytes, etc. These effector cells play an important role in phagocytosis, cytokine production, antigen presentation, and the release of inflammatory mediators. The adaptive immune systems mainly act on specific antigens and stimulate B cells and T cells to produce antibodies [19]. Ginseng was chosen as the positive group because its bioactive components can improve efficacy while reducing the toxicity of cyclophosphamide in cancer treatment [20].

In this study, we investigated the immunomodulatory effects and mechanisms of Maca polysaccharides in an immunosuppression model by observing the impacts of Maca polysaccharides on animal behaviours and functions.

## 2. Materials and Methods

### 2.1. Materials and Reagents

*Lepidium meyenii Walp* (Maca) root powder was provided by New Era Health Industry (Group) Co. Ltd. (Beijing, China); the lot number was 20150315. Ginseng granules were obtained from Beijing Tcmages Pharmaceutical Co., Ltd., and the lot number was 17006312. One gram of ginseng granule equals five grams of ginseng pieces. An inspection report of ginseng was provided; the sum of ginsenoside Rg1 and ginsenoside Re was 0.55%, and the content of ginsenoside Rb1 was 0.74% per gram of ginseng granule. We purchased injectable cyclophosphamides from Bioway Co., Ltd. (Shanghai, China). IFN-*γ* ELISA kits (cat: HY-2447), TNF-*α* ELISA kits (cat: HY-H0019), IL-2 ELISA kits (cat: HY-H0003), and IL-4 ELISA kits (cat: HY-10103) were all purchased from Sino-UK (Beijing, China).

For the preparation of Maca root polysaccharide extract (MP), Maca root powder was extracted twice with water at a solid-liquid ratio of 1 : 20 (mg/ml) at 70°C for 1.5 h. Then, the water extraction was purified with alcohol and resin (ion-exchange column and gel filtration chromatography). The Maca polysaccharide extract yield reached 9.88 mg/g, and the total sugar and protein purity were 75.42% and 7.73%, respectively, with further isolation.

### 2.2. Establishment of an Immunosuppressed Mouse Model and Treatment Protocols

**Figure 1:**
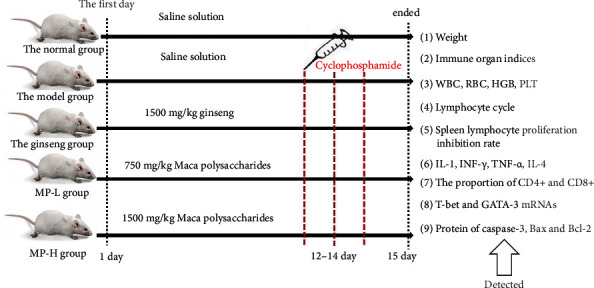
Flow chart of the experiment procedure.

Sixty healthy Kunming male mice (weighing 20 ± 2 g) were purchased from SPF (Beijing) Biotechnology Co. Ltd., and the permit number is SCXK (Beijing) 2016-0002. The trials complied with the health *Guide for the Care and Use of Laboratory Animals* (Ministry of Science and Technology of China, 2006) and were approved by the Ethics Committee of Beijing University of Chinese Medicine (BUCM-4-2017081220-3020). The mice were kept in the SPF Animal Feeding Center (12 h light/12 h dark cycle, temperature of 23 ± 2°C, humidity of 60% ± 5%), which belongs to Beijing University of Chinese Medicine. The mice were fed normal feed and water. After one week of acclimatization, sixty mice were randomly divided into 5 groups, including the normal group, the model group, the positive group, the low-dose Maca polysaccharide group (MP-L), and the high-dose Maca polysaccharide group (MP-H), and each group had 12 mice. The experiment lasted for 14 days. In the experimental process, saline solution was orally administered to the normal group mice and the model group mice; the positive control group mice were given ginseng (1500 mg/kg); the MP-L group mice were given Maca polysaccharides (750 mg/kg); the MP-H group mice were given Maca polysaccharides (1500 mg/kg). All the groups were intragastrically administrated for the prevention, respectively, once every day continuously for 14 days from the first day. Except for the normal group, the other groups were intraperitoneally injected with cyclophosphamide (60 mg/kg) on the 12th to 14th days to establish a model (Figure 1).

### 2.3. The Body Weight and Immune Organ Indices

We must observe the general health conditions of mice from appearance, activeness, and feeding behaviour closely during the experiment. On Days 0, 5, 11, and 14, each group of mice was weighed, and the data was recorded. After the treatment period, the mice were sacrificed, and the spleen and thymus were immediately excised surgically and weighed to calculate the spleen and thymus indices. We calculated the thymus and spleen indices according to the following formula:

(1) $$\begin{array}{c} \text{Index }\left(\text{mg}/\text{g}\right)=\frac{\text{weight of thymus or spleen}}{\text{body weight}}. \end{array}$$

### 2.4. Haematological Parameters

Whole blood was obtained by removing eyeballs and then collected in clean test tubes with ethylenediaminetetraacetic acid (EDTA). A proper amount of whole blood was used to detect the counts of white blood cells (WBCs), red blood cells (RBCs), haemoglobin (HGB), and platelets (PLTs) by a Beckman Coulter Ac and T 5 full-automatic blood cell analyser (Beckman, USA).

### 2.5. Peripheral Blood Lymphocyte Cycle

Whole blood was collected into tubes containing heparin sodium and then made into peripheral blood lymphocyte suspension. The cell suspension was transferred and centrifuged at 1500 rpm for 5 min to collect peripheral blood lymphocytes. Then, peripheral blood lymphocytes were washed twice with PBS. The prepared single-cell suspension was stored at 4°C and washed with PBS before staining the fixing solution. RNAse was added to a 37°C water bath for 40 min and added propidium iodide (PI) staining and mixed well, and then, it was incubated at 4°C for 30 min. Finally, flow cytometry was used to detect the fluorescence of the PI-DNA complex.

### 2.6. MTT Assay

The spleen was aseptically removed from the mice, and spleen lymphocytes were prepared. Spleen lymphocyte viability was determined by the MTT method. The spleen lymphocyte suspension (1 × 10^4^‐5 × 10^4^ cells/mL) was placed into 96-well microplates at 37°C in an incubator with 5% CO_2_. We added MTT (5 mg/ml) to each well and then incubated the cells for 4 h at 37°C. After incubation, we discarded the medium and subsequently dissolved the formazan crystals with 150 *μ*L of DMSO. We used a Molecular Devices Spectra Max M5 (Molecular Devices, USA) to measure the absorbance of the solution at a wavelength of 490 nm. At the same time, we set up the blank group and the negative control group, the black control group did not contain any cells, and the negative control group contained normal spleen lymphocytes without drug treatment. Other operations in these two groups were the same as in the treated group. To determine the spleen lymphocyte viability inhibition rate, the following equation was used: inhibition rate (IR) = 100% − (*A* − *B*)/(*C* − *B*) × 100%, where *A* was the absorbance of the treated groups, *B* was the absorbance of the blank control group, and *C* was the absorbance of the negative control group.

### 2.7. Flow Cytometric Analysis

Flow cytometry was used to measure the proportion of CD4+ and CD8+ lymphocyte subtypes in peripheral blood in each group, and we calculated the ratio of CD4+/CD8+. Peripheral blood (100 *μ*l) was placed in EDTA in a labelled tube, and 20 *μ*l of fluorescent anti-CD3, anti-CD4, and anti-CD8 monoclonal antibodies was added at 37°C for 20 min in the dark. After haemolysis, samples were centrifuged for 10 min at 1500 rpm at room temperature, washed twice in PBS, and subjected to flow cytometry (BD Biosciences, Franklin Lakes, NJ, USA) to analyse T lymphocyte subsets (CD4+ and CD8+). The percentage of CD4+ and CD8+ T cells was determined using Cell-Quest software (Beckman Coulter).

### 2.8. ELISAs

After the last day of treatment, whole blood was drawn into tubes from mice by eyeball extraction and centrifuged at 4°C and 3500 r for 15 min. The supernatant was aspirated and frozen at -20°C until use. The contents of IFN-*γ*, TNF-*α*, IL-2, and IL-4 in serum were quantitatively detected by ELISA kits, and the manufacturer's instructions were followed.

### 2.9. Quantitative Real-Time PCR (RT-qPCR)

The changes in T-bet and GATA-3 mRNAs were explored by a quantitative real-time PCR assay. First, total RNA was extracted with a TRIzol reagent kit (Invitrogen, Carlsbad, USA) and tested for concentration and purity. Then, the RNA was reverse transcribed into cDNA by a Super RT cDNA kit. SYBR® Green PCR Master Mix was used to amplify cDNA in the Multicolour Real-time PCR Detection System. In addition, the sequences for primers are listed in Table 1. The PCR parameters were as follows: 95°C for 10 min, followed by 40 cycles of 60°C for 15 s, 75°C for 1 min, and 95°C for 15 s. Temperature increases were 1°C per 20 s. RT-qPCR analysis was performed with the Light Cycler 480 RT-qPCR System (Roche, Basel, Switzerland). T-bet and GATA-3 mRNA levels were normalized to the endogenous reference GAPDH, and the results were calculated by the 2−*ΔΔ*Ct method.

### 2.10. Western Blot Analysis

The total proteins of the spleen were extracted from a standard lysis buffer containing proteinase inhibitors, and then, the protein concentration in the lysates was determined by a BCA protein assay kit (cat: G2026, Servicebio, Wuhan, China). By denaturing the proteins by the boiling method, equal amounts of proteins were separated by 10% sodium dodecyl sulfate-polyacrylamide gel electrophoresis (SDS-PAGE) and transferred onto a polyvinylidene difluoride (PVDF) membrane. The membranes were blocked with nonfat milk (5%) for 1 h at room temperature and incubated with anti-Bax rabbit pAb (cat: GB11690), anti-caspase-3 rabbit pAb (cat: GB11767), anti-Bcl-2 mouse mAb (cat: GB12318), and mouse anti-*β*-actin antibody (cat: GB12001) at 4°C overnight. All of the antibodies were purchased from Servicebio (Wuhan, China). After washing three times for 15 min, the membrane was incubated with HRP-conjugated secondary antibodies. Finally, the blots were visualized by an enhanced chemiluminescence (ECL) kit (cat: G2014, Servicebio, Wuhan, China).

### 2.11. Statistical Analysis

All statistical analyses were performed using SPSS (version 22.0, SPSS Inc., Chicago, IL, USA), and data are expressed as the mean ± standard deviation (*x* ± *s*). One-way ANOVA followed the least-significant difference (LSD) post hoc test or Dunnett T3 test for comparisons of multiple groups. Significant differences were set at *p* < 0.05.

## 3. Results

### 3.1. The Health, Body Weight, and Immune Organ Indices

We observed that the model mice exhibited disheveled fur, less movement, and crouching symptoms after injection of CYP. But the mice treated with ginseng and MP had significantly improved.

**Figure 2:**
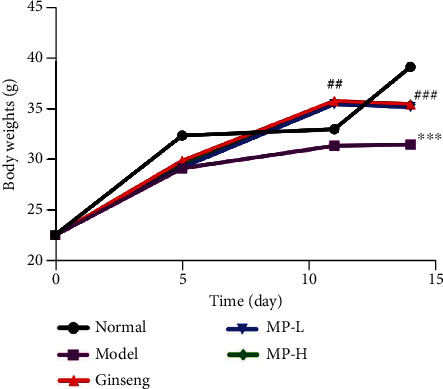
Body weights of mice (*n* = 12). Notes: ^∗∗∗^*p* < 0.001 vs. control group; ^#^*p* < 0.05 and ^##^*p* < 0.01 vs. model group.

As shown in Table 2 and Figure 2, before injection with CYP, the body weights of each group were basically the same. After injection with CYP, compared with the normal group, the body weights of model mice were significantly decreased (<0.001). However, the body weights of mice in the ginseng, MP-L, and MP-H groups showed no significant declines. Compared to the model group, the mice treated with ginseng and MP both had an increase in weight (<0.01 or <0.001).

**Figure 3:**
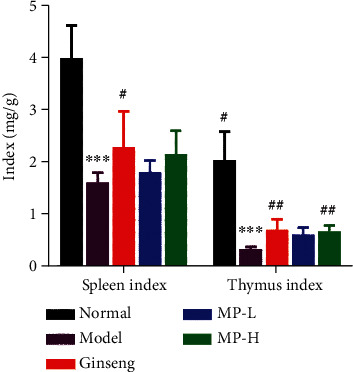
Indices of spleen and thymus (*n* = 12). Notes: ^∗∗∗^*p* < 0.001 vs. control group; ^#^*p* < 0.05 and ^##^*p* < 0.01 vs. model group.

Thymus and spleen indices can reflect the immune function of the body to a certain extent. As shown in Figure 3, after CYP treatment, the thymus and spleen were obviously atrophied, and their indices both decreased significantly in the normal group (<0.001). Compared to the model group, the spleen index (<0.05) and thymus index (<0.01) significantly increased in the MP-H and ginseng groups but did not return to normal levels.

### 3.2. Haematological Parameters

**Figure 4:**
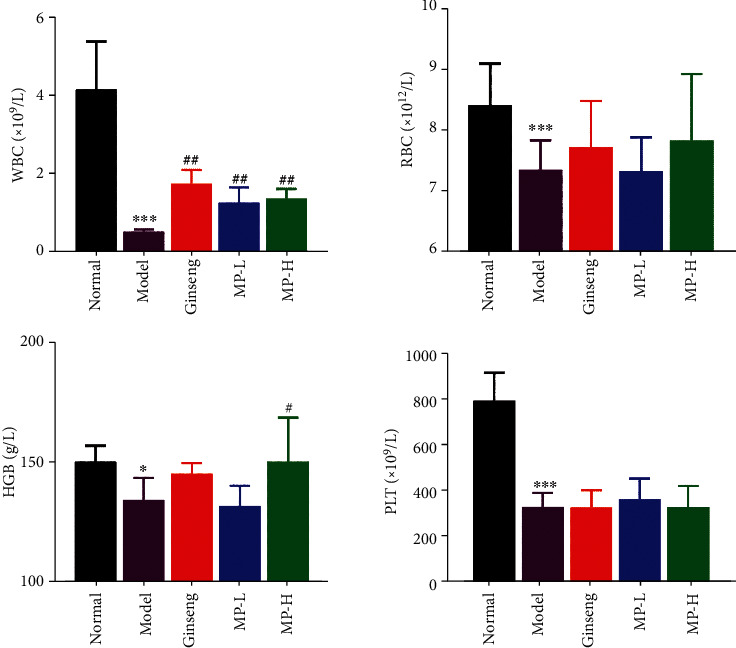
Effects of Maca polysaccharides on haematological parameters (*n* = 12); ^∗^*p* < 0.05 and ^∗∗∗^*p* < 0.001 vs. control group; ^#^*p* < 0.05 and ^##^*p* < 0.01 vs. model group.

To investigate the activities of MP in abnormal haemograms, the classical experimental model of immunosuppressed mice was injected with CYP. As depicted in Figure 4, compared with the normal group, the peripheral blood cells, including white blood cells, red blood cells, and platelets, all decreased significantly following injection of CYP (<0.001), and the haemoglobin counts in blood also declined (<0.05) in the model group. There were obvious changes in the counts of WBCs and HGB compared to the model group. Ginseng and MP significantly increased WBC counts (<0.01) without returning to normal levels, and only the high dose of MP had an effect on the elevation of HGB (<0.05). There were no significant differences in RBC counts and platelet counts in the groups given ginseng and MP when compared to the model group.

### 3.3. Lymphocyte Subsets of Peripheral Blood

**Figure 5:**
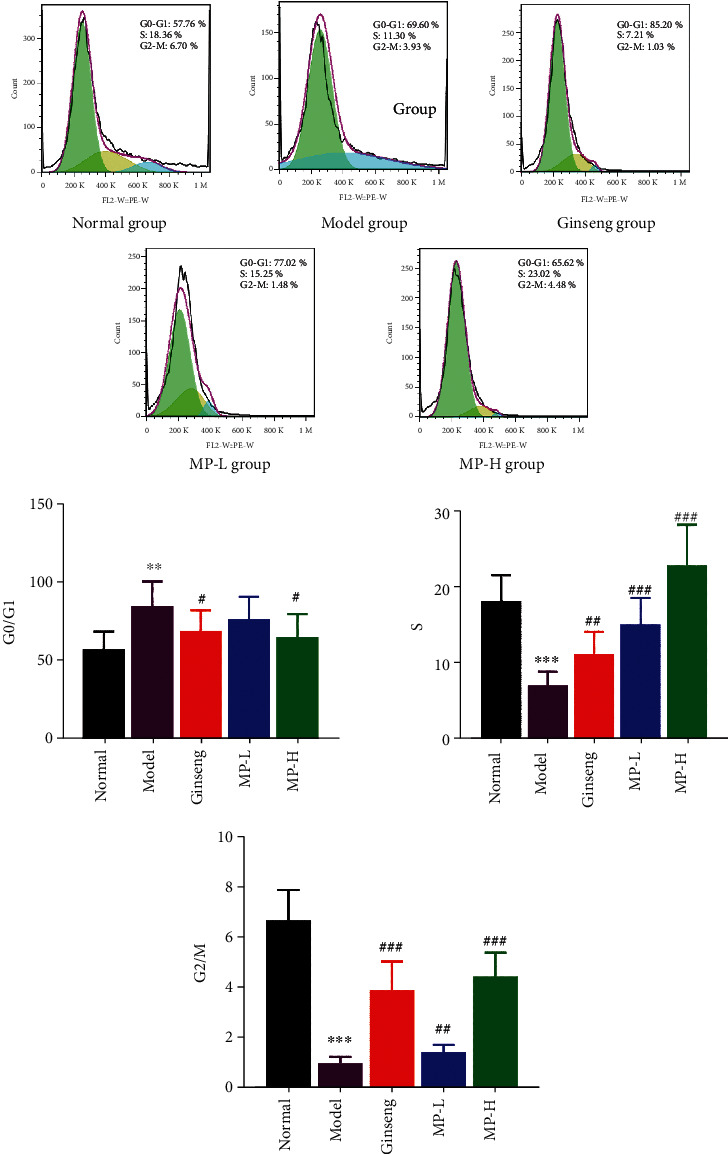
Effects of Maca polysaccharides on the peripheral blood lymphocyte cycle (*n* = 12); ^∗∗^*p* < 0.01 and ^∗∗∗^*p* < 0.001 vs. control group; ^#^*p* < 0.05, ^##^*p* < 0.01, and ^###^*p* < 0.001 vs. model group.

Peripheral blood lymphocytes were collected, and the flow cytometry method was used to observe the percentage of lymphocyte cycles in each group (Figure 5). The results showed that the proportion of peripheral blood lymphocytes remaining in the G0/G1 phase increased significantly (<0.01) after CYP injection. The ginseng group and MP-H group showed modest reductions in the proportion of lymphocytes in the G0/G1 phase (<0.05). Compared with the normal group, the percentage of lymphocytes staying in the S phase and the G2/M phase decreased (<0.001). Compared with the model group, both ginseng and MP significantly increased the percentages of the S phase and G2/M phase of the peripheral blood lymphocyte cycle (<0.01 or <0.001) (Figure 5).

**Figure 6:**
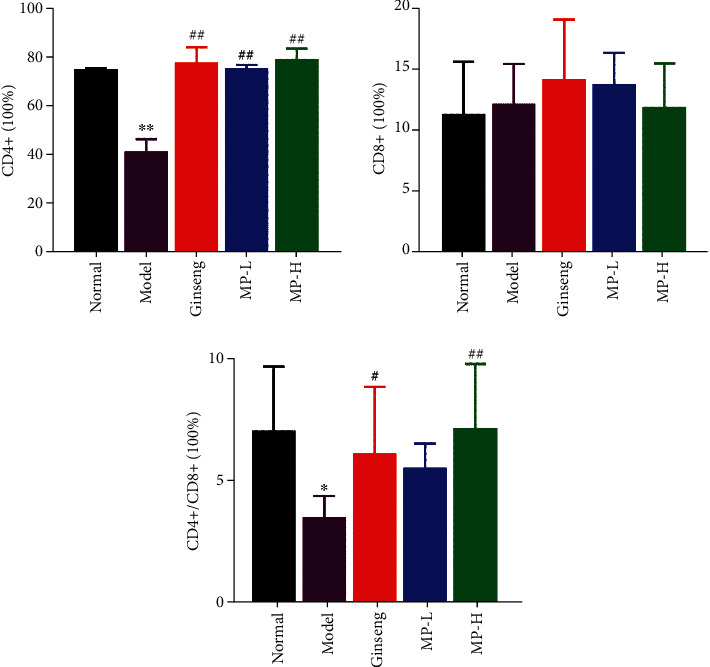
Effects of Maca polysaccharides on CD4+ T cells and CD8+ T cells (*n* = 12); ^∗^*p* < 0.05 and ^∗∗^*p* < 0.01 vs. control group; ^#^*p* < 0.05 and ^##^*p* < 0.01 vs. model group.

After the separation of CD4+ T cells and CD8+ T cells, their relative levels were examined by flow cytometry. From the results in Figure 6, CYP significantly inhibited the proliferation of CD4+ T lymphocytes (<0.01) compared to the normal group, and the ratio of CD4+/CD8+ declined intensely (<0.05), although the percentage of CD8+ cells was not obviously different between the normal group and the model group. Compared with the model group, the percentage of CD4+ T cells increased to a normal state in all administration groups (<0.01), but only ginseng and the high dose of MP had effects on increasing the ratio of CD4+/CD8+ (<0.01 or <0.05). Therefore, through the data just described, it was suggested that the immunomodulatory effect of Maca is manifested by regulating the percentage number of CD4+ T lymphocytes and CD8+ T lymphocytes.

### 3.4. Spleen Lymphocyte Inhibition Rate

**Figure 7:**
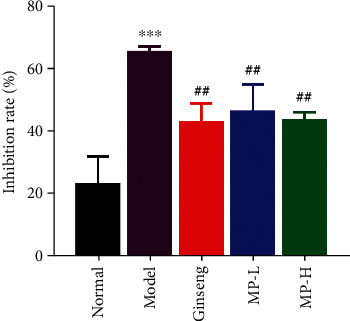
Effects of Maca polysaccharides on spleen lymphocyte inhibition rate (*n* = 12); ^∗∗∗^*p* < 0.001 vs. control group; ^##^*p* < 0.01 vs. model group.

We investigated the viability of spleen lymphocytes using an MTT assay. As shown in Figure 7, cyclophosphamide drastically decreased the survival rate of spleen lymphocytes (<0.001), and ginseng and MP reversed this decline in survival significantly (<0.01) without returning to normal levels.

### 3.5. Cytokine Levels in Serum and Spleen Tissue

**Figure 8:**
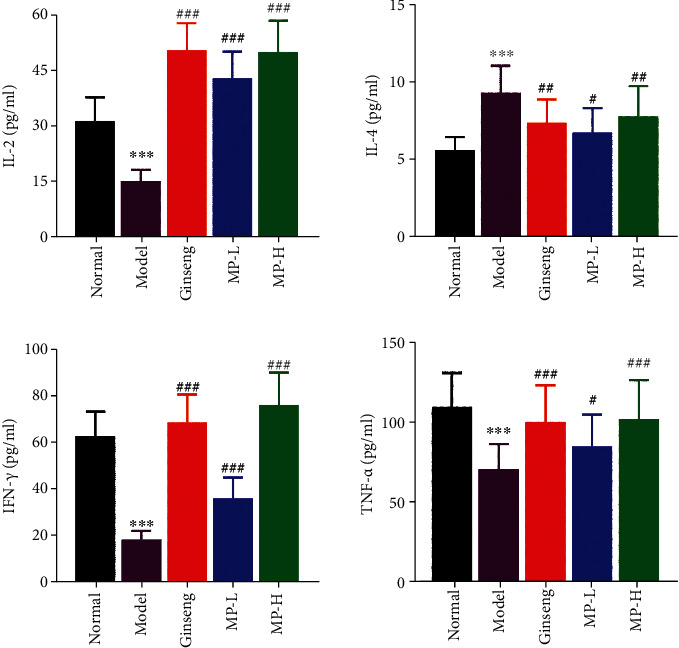
Effects of Maca polysaccharides on serum cytokines of IFN-*γ*, TNF-*α*, IL-2, and IL-4 (*n* = 12); ^∗∗∗^*p* < 0.001 vs. control group; ^#^*p* < 0.05, ^##^*p* < 0.01, and ^###^*p* < 0.001 vs. model group.

We assessed the immunomodulatory function of Maca polysaccharides by detecting the secretion of cytokine. At the end of the experimental period, the positive factors IL-2, IFN-*γ*, and TNF-*α* and the negative factor IL-4 in immunosuppressed mice were analysed. As shown in Figure 8, the model group secreted lower levels of IL-2, IFN-*γ*, and TNF-*α* (<0.001) than the normal group, but the production of IL-4 increased rapidly (<0.001) at the same time. In comparison to the model group, the counts of serum cytokines of IL-2, IFN-*γ*, and TNF-*α* were drastically increased (<0.05 or <0.001), and IL-4 rapidly decreased (<0.05 or <0.01) in all administration groups.

**Figure 9:**
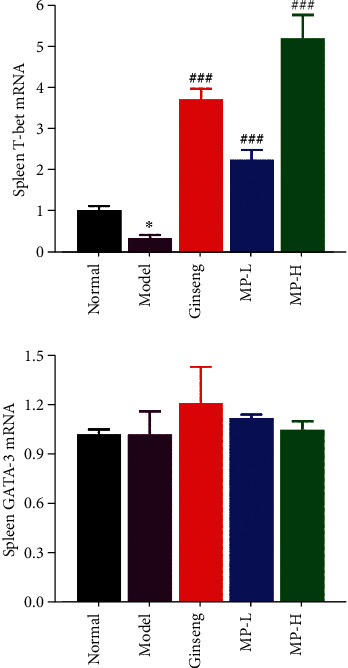
Effects of Maca polysaccharides on the mRNA expression of spleen T-bet and GATA-3 (*n* = 12); ^∗^*p* < 0.05 vs. control group; ^##^*p* < 0.01 and ^###^*p* < 0.001 vs. model group.

T-bet and GATA-3 are key factors that specifically regulate the differentiation of Th0 cells into Th1 and Th2 cells. T-bet is a Th1-specific transcription factor that plays an important role in the development of Th1 cells and inhibits the synthesis of Th2 cytokines. GATA-3 is the opposite of T-bet. We evaluated the Th1/Th2 balance by detecting the mRNA expression of T-bet and GATA-3 in the spleen. From the results in Figure 9, the mRNA expression of T-bet was reduced sharply in the model group (<0.05), but the level of T-bet mRNA was increased in all treatment groups (<0.001). The GATA-3 mRNA levels in each treatment group were slightly higher than those in the model group, although there were no significant differences. Therefore, the balance between T-bet and GATA-3 is broken, which means that the equilibrium between Th1 and Th2 cells is out of balance.

### 3.6. Protein Expression of Factor-Related Apoptosis in Spleen

**Figure 10:**
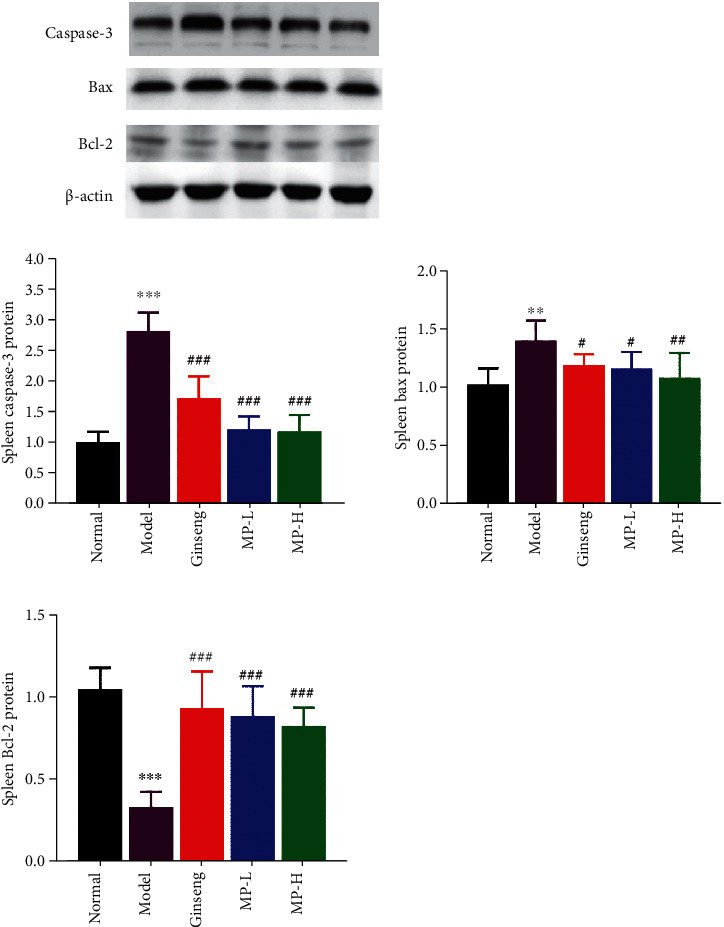
Protein expression of caspase-3, BAX, and the Bcl-2 in the spleen (*n* = 3); ^∗∗^*p* < 0.01 and ^∗∗∗^*p* < 0.001 vs. control group; ^#^*p* < 0.05, ^##^*p* < 0.01, and ^###^*p* < 0.001 vs. model group.

Caspase-3, BAX, and Bcl-2 are related to apoptosis. Caspase-3 and BAX are positive factors that could promote apoptosis, and Bcl-2 is a negative factor for apoptosis. We used the Western blot method to detect the protein expression levels of caspase-3, BAX, and Bcl-2 in the spleen, and the results are shown in Figure 10. Compared to the normal group, the caspase-3 and BAX protein levels increased acutely (<0.01 or <0.001), and the Bcl-2 protein contents decreased (<0.001) in the model group. Caspase-3 and BAX protein levels were modestly reduced in mice gavaged with MP or ginseng (<0.05 or <0.01 or <0.001), which was significantly different from the model group. The expression of Bcl-2 protein was increased in all treatment groups compared to the model group (<0.001).

## 4. Discussion

Cyclophosphamide has been extensively used in oncology as a chemotherapeutic agent, but it could cause serious adverse effects on normal cells when it inhibits the proliferation of cancer cells [21]. The most noticeable side effect caused by cyclophosphamide is immunosuppression, which may cause clinical manifestations such as leukopenia, loss of appetite, nausea, vomiting, fatigue, and other adverse reactions. These clinical manifestations are relatively similar to spleen deficiency in TCM. Spleen deficiency syndrome (SDS) is a typical syndrome of traditional Chinese medicine (TCM) and is characterized by poor appetite, fullness, sleepiness after eating, nausea, vomiting, fatigue, pale face and tongue, weight loss, and loose stools [22, 23]. In traditional Chinese medicine (TCM), “spleen” is completely different from that of the spleen organ in Western medicine. From the perspective of modern medicine, “spleen dysfunction” in TCM is mostly associated with energy metabolism and the immune system. Previous studies have shown that the energy metabolism and immune system of the body could decrease when the spleen is weak, and Maca powder has an effect on the function of immunity and energy metabolism for spleen deficiency syndrome [24]. Therefore, this research associates the TCM spleen with immunoregulation to explore the immunoregulatory mechanism of Maca polysaccharides based on a spleen deficiency model.

The function of immunomodulation of the body depends on the normal function of immune organs, immune cells, and immune factors. The thymus and spleen are important immune organs, and their organ index can directly reflect the level of immune functions of the body [19]. The effects of drugs on the spleen and thymus index can be used as preliminary indicators for the study of immune pharmacological mechanisms in animals. In this study, the body weight and organ index in the model group were decreased, while weight loss was reversed in mice in both the Maca polysaccharide and ginseng groups. High-dose Maca polysaccharides could promote the spleen and thymus index simultaneously. These results suggested that Maca polysaccharides have a positive role in preventing body weight loss and could strengthen immune function through spleen function.

Myelosuppression induced by cyclophosphamide administration is the most common complication in clinical chemotherapy. Previous studies have shown that cyclophosphamide treatment could cause a suppressive effect on haematological parameters [25] and markedly decreased the count of peripheral RBC, WBC, HGB, and platelets [26]. In our study, the administration of Maca polysaccharides and ginseng increased the counts of WBCs and haemoglobin, which was significantly lowered by CYP but had no significant effect on RBCs or platelets. The haematological parameters indirectly reflect the condition of bone marrow haematopoiesis, and our results signify the immunomodulatory potential of Maca polysaccharides against cyclophosphamide-induced myelosuppression.

T lymphocytes can be divided into four types: CD4+ helper T cells (Th cells), CD8+ cytotoxic T cells (CTLs), T suppressor cells, and T effector cells according to their functions and surface markers [27]. CD4+ T helper cells and CD8+ cytotoxic T cells participate in the immune response process together, and the count of CD4, CD8 lymphocytes, and the ratio of CD4/CD8 changes are important factors in pathogenesis. When the body is in a state of severe disease or poor prognosis, the ratio of CD4+/CD8+ decreases, but the ratio increases when the body is in a state of positive regulation of the immune response. Therefore, they are usually used to evaluate the body's immune function. In the present study, Maca polysaccharides antagonized the immunosuppressive effects of cyclophosphamide by increasing the CD4 lymphocyte count and the CD4+/CD8+ subset ratio. These findings indicated that Maca polysaccharides can exert immunomodulatory function by regulating T lymphocyte subsets.

CD4+ helper T cells (Th cells) can differentiate into Th1 and Th2 cell subtypes depending on the type of cytokine production and biological function [28–30]. Th1 cells induce inflammatory responses and support the immune system in fighting intracellular pathogens [31, 32], whereas Th2 cells primarily aid the differentiation of B cells into antibody-producing plasma cells [33]. In the physiological state, Th1 cells and Th2 cells maintain a dynamic equilibrium state, but the Th1/Th2 ratio balance is disrupted when the immune regulation function is damaged. T-bet and GATA-3 are important transcription factors that affect the differentiation of Th1 and Th2 cells [34]. Th1 cells can secrete positive regulators such as IFN-*γ*, TNF-*α*, and IL-2 [35, 36], and IL-4 secreted by Th2 cells is a negative factor [37]. In addition, IFN-*γ* and IL-4 are a pair of important immune cytokines that antagonize each other [38]. T-bet is a powerful transcriptional activator of IFN-*γ*, which can induce Th0 cells to differentiate into Th1 cells and inhibit Th2 cell differentiation; in contrast, GATA-3 can improve the transcriptional activity of the promoter of IL-4, inhibit the expression of IFN-*γ*, induce T0 cells to differentiate into T2 cells, and inhibit T1 cell differentiation [39]. Therefore, the imbalance of T-bet/GATA-3 mRNA expression is closely related to the imbalance of Th1/Th2 and could be used as a new indicator to measure Th1/Th2 balance [40]. The results showed that Maca polysaccharides can transform Th0 cells into Th1 cells and inhibit the activation of Th2 cells by regulating the mRNA expression of T-bet and GATA-3 in spleen cells and by maintaining the Th1/Th2 ratio balance to promote immune balance homeostasis. In addition, cytokines play a key role in regulating the immune system and can directly or indirectly regulate immune reactions. For example, IL-2 can enhance the vitality of NK cells, cytotoxic T cells, monocytes, and macrophages and improve the proliferation and secretion of antibodies by B lymphocytes [41]. In our study, Maca polysaccharides significantly upregulated the expression of IFN-*γ*, IL-2, and TNF-*α* but decreased the levels of IL-4 in the serum. The results indicated that the enhancement of Th1 cytokines and the decrease in Th2 cytokines caused by Maca polysaccharides may explain the immune response.

It is generally recognized that cell cycle arrest usually indicates decreased cell viability or even cell apoptosis [42]. To further study the effect of Maca polysaccharides on the immunosuppressive mouse model, peripheral blood lymphocyte cycle analysis was performed. The results showed that the lymphocyte cycle stops in G0/G1 phase after mice are injected with cyclophosphamide, but Maca polysaccharides could reduce the ratio of lymphocytes in the G0/G1 phase and increase the percentage of S phase and G2/M phase, which reveals that Maca polysaccharides could promote lymphocytes to enter the S phase and the G2/M phase.

Lymphocyte proliferation is related to cellular and humoral immune responses that protect against infection by foreign antigens [43]. T lymphocytes are associated with cell-mediated immune responses, while B lymphocytes and antibodies secreted from plasma cells are the key elements involved in the humoral immune response [44]. By examining the survival rate of spleen lymphocytes in the presence of polysaccharides, the effects of polysaccharides on the proliferation of spleen lymphocytes can be analysed. Our results showed that Maca polysaccharides significantly reversed the survival rate of spleen lymphocytes.

The apoptosis of immune cells is closely related to the regulation of the immune system, and it is an important index to evaluate the changes in immune function [45]. As important regulators of apoptosis, the expression of both antiapoptotic proteins represented by Bcl2 and proapoptotic proteins represented by Bax and caspase-3 determine the trend of apoptosis [46, 47]. Therefore, we studied the contents of antiapoptotic proteins and proapoptotic proteins in the spleen to illustrate the body's immune system. Our results showed that Maca polysaccharides could upregulate the expression of Bcl2 and downregulate the expression of Bax and caspase-3 in spleen tissue, which confirmed that Maca polysaccharides could decrease the apoptosis of spleen lymphocytes in immunosuppression model mice.

## 5. Conclusion

**Figure 11:**
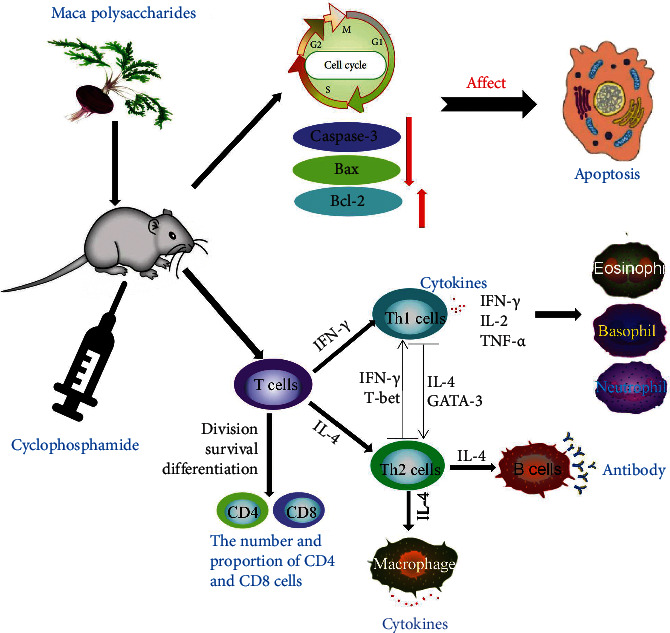
The mechanism of this study.

In this study, Maca polysaccharides were obtained from Maca, and their immunomodulatory effect on an immunosuppressive model induced by cyclophosphamide was studied. The results showed that Maca polysaccharides could improve the general state of the model, increase the body weight and organ indices, and regulate the activity of immune cells and the secretion of cytokines (Figure 11). All the data in this study provide evidence that Maca polysaccharides show an obvious immunomodulatory effect on immunosuppressive syndrome, which indicates that they are an important material basis of Maca. Furthermore, our findings indicate that Maca polysaccharides have potent immunomodulatory properties and might be considered a novel potential immunomodulator for use in drugs or functional foods.

## Figures and Tables

**Table 1:** Primers used for quantitative RT-qPCR.

Genes	Forward (5′–3′)	Reverse (5′–3′)
T-bet	ACTAAGCAAGGACGGCGAATG	GTCCACCAAGACCACATCCACA
GATA-3	GAACTGCGGGGCAACCTCTA	GCCTTCGCTTGGGCTTGATA
GAPDH	CCTCGTCCCGTAGACAAAATG	TGAGGTCAATGAAGGGGTCGT

**Table 2:** Body weights of mice (g; *x* ± *s*; *n* = 12).

Groups	0 day	5th day	11th day	14th day
Normal	22.63 ± 1.30	32.26 ± 1.40	32.99 ± 2.76	39.10 ± 2.73
Model	22.60 ± 1.19	29.15 ± 3.12	31.37 ± 4.44	31.48 ± 3.74^∗∗∗^
Ginseng	22.60 ± 1.10	29.88 ± 2.15	35.76 ± 3.08^##^	35.44 ± 3.44^##^
MP-L	22.60 ± 1.07	29.24 ± 1.57	35.44 ± 2.31^##^	35.16 ± 2.57^##^
MP-H	22.60 ± 1.04	29.48 ± 1.35	35.57 ± 2.73^##^	35.41 ± 2.92^###^

Notes: ^∗∗∗^*p* < 0.001 vs. control group; ^##^*p* < 0.01 and ^###^*p* < 0.001 vs. model group.

## Data Availability

The data (original) used to support the findings of this study are available from the authors upon request.
